# Treatment of osteoblastoma at C3-4 in a child: a case report

**DOI:** 10.1186/1471-2474-15-313

**Published:** 2014-09-26

**Authors:** Ganjun Feng, Kangkang Huang, Li Li, Quan Gong, Hao Liu, Yueming Song

**Affiliations:** Department of Orthopedics, West China Hospital, Sichuan University, 17 Renmin South Road, Chengdu, Sichuan (610041) P.R. China; Department of Health Examination Center, West China Hospital, Sichuan University, Chengdu, Sichuan (610041) P.R. China

**Keywords:** Osteoblastoma, C3-4, Child, Vertebral artery injury

## Abstract

**Background:**

Osteoblastoma is a rare and benign osteoid-producing primary bone tumor that affects mainly the long bones. 36% of these tumors are observed around the spine and the vast majority arises around the posterior.

**Case presentation:**

This report describes a case of C3-4 osteoblastoma occurring in a 5-year-and-8-month-old Han Chinese child. The pathophysiology of symptom development, evaluations, and management are presented. Because of the close proximity of the osteoblastoma to the vertebral artery canal, the artery suffered a minor laceration intraoperatively. Hemostatic gelatin sponges were used to compress the bleeding site instantly and a tricortical iliac crest fixed with a screw was also used to add pressure to the gelatin sponges. Fusion on the other side was also used to stabilize the spine. To the best of our knowledge, this is the first report of a case of osteoblastoma at C3-4 with artery injury intraoperatively.

**Conclusions:**

This case delineates the difficulties in diagnosing this tumor, the challenges and problems encountered during its surgical management, and the favorable prognosis after adequate treatment.

**Electronic supplementary material:**

The online version of this article (doi:10.1186/1471-2474-15-313) contains supplementary material, which is available to authorized users.

## Background

Osteoblastoma is a rare and benign osteoid-producing primary bone tumor that affects mainly the long bones. Thirty-six percent of these tumors are observed around the spine and the vast majority arises around the posterior [1, 2]. The mean age at presentation was 20.4 years, with a range of 6 months to 75 years [3]. Several cases of osteoblastoma of the cervical vertebrae have been reported and different aspects of their management have been discussed [4–7]. However, cases occurring at C3 or C4 are rare. This report describes a case of C3-4 osteoblastoma occurring in a 5-year-and-8-month-old child. The pathophysiology of symptom development, evaluations, and management are also presented.

## Case presentation

A Han Chinese boy aged 5 years and 8 months was admitted to the hospital for evaluation of neck and right shoulder and arm pain persisting for more than 6 months. The intensity of the pain had gradually increased during the course of the illness. However, there was no pain during sleep.

**Figure 1:**
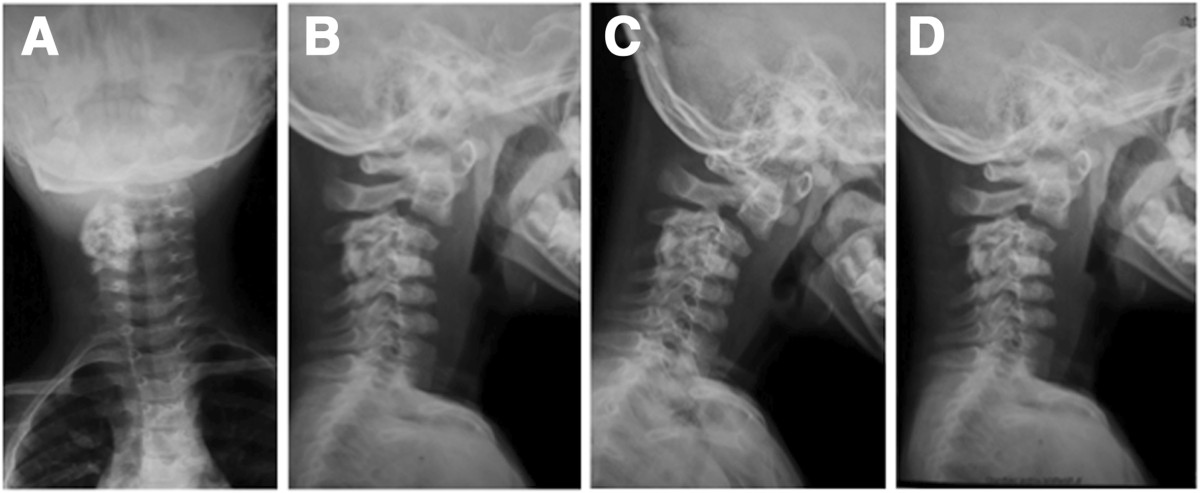
**Preoperative radiographs. (A)**: Anteroposterior view shows an oval high-intensity area and a well-defined rim from C3 to C4 in the right side. **(B)**: Lateral radiograph shows a lesion arising from the posterior arch of C3-4. **(C)** and **(D)**: Postoperative anteroposterior and lateral plain radiographs show the tumor has been removed grossly and internal fixation has been performed.

**Figure 2:**
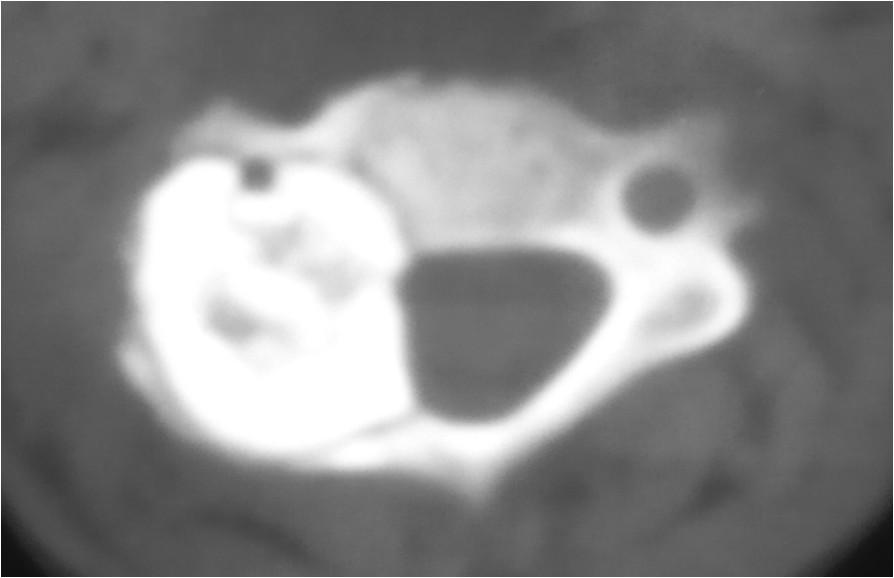
**Computed tomography indicates the presence of a massive tumor from right lamina of C3 and extending to the spinal canal.** The tumoral mass circumference has a sclerosis band, and the inhomogeneous matrix ossification is presented in the lesion. The canal of vertebral artery is also encroached by the lesion.

**Figure 3:**
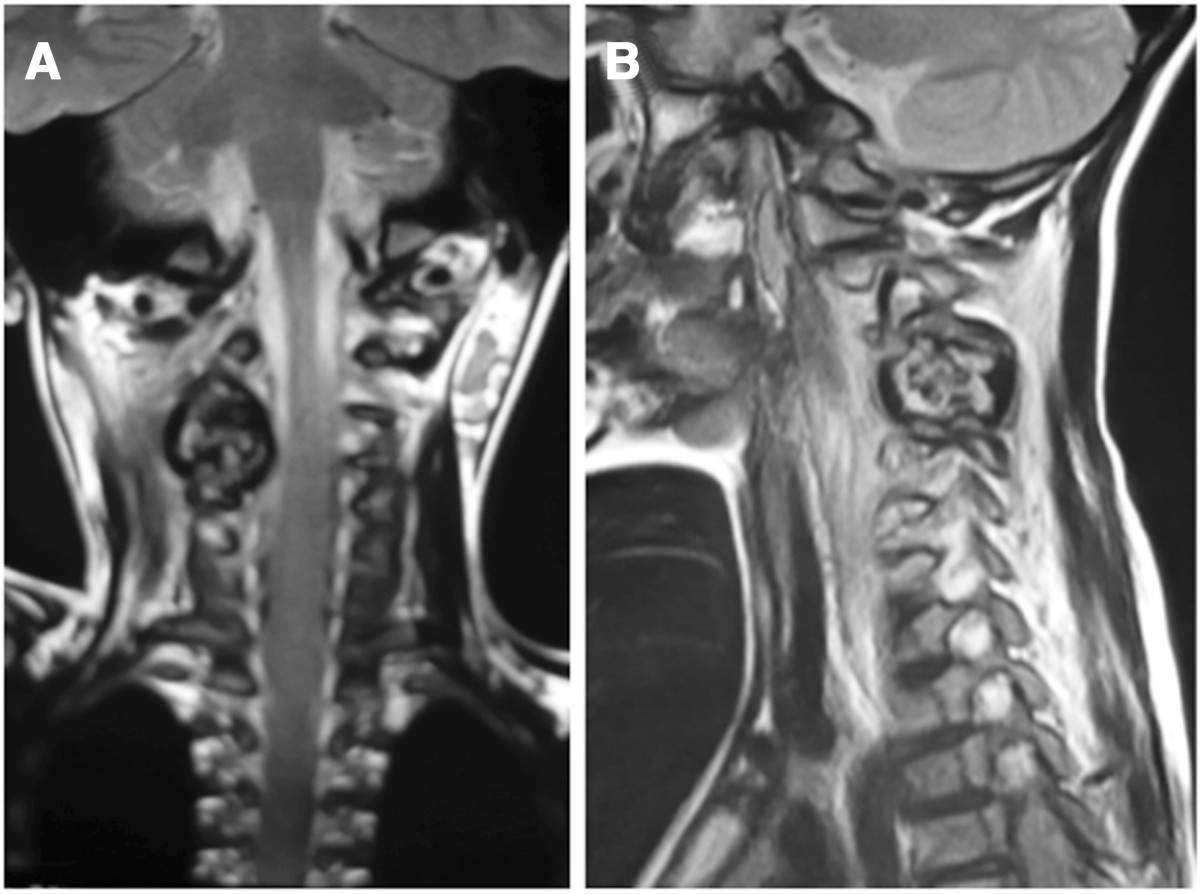
**The tumor contained a low-intensity center in T1W (A) and high-intensity center in T2W images (B), combined with intermedial and outer lesion matrix ossification signals.**

On physical examination, the patient tended to keep his neck slightly bent and twisted to the right side, and the pain aggravated severely with motion. The strength of the muscle groups of his right hand was Grade 4/5, while the strength of the other muscle groups was normal. His right-side Hoffmann’s sign and the Babinski’s sign for both sides were positive. All deep tendon reflexes were also exaggerated. No sensory changes were noted on physical examination.Plain radiographic studies showed an oval radiolucent lesion located on the pedicle and facet of C3-4 vertebrae on the right side in the anteroposterior (Figure 1A) and lateral views (Figure 1B). Flexion and extension radiographs showed no spinal instability (Figure 1C, D). A computed tomography (CT) scan showed a massive tumor extending from right lamina of C3-4 to the facet and pedicle. The lesion encroached upon the radicular foramina and was closely adjacent to the canal of the vertebral artery (Figure 2). On magnetic resonance imaging, the tumor presented a low-intensity center in T1W and a high-intensity center in T2W images, combined with intermedial and outer lesion matrix ossification signals (Figure 3A, B).

**Figure 4:**
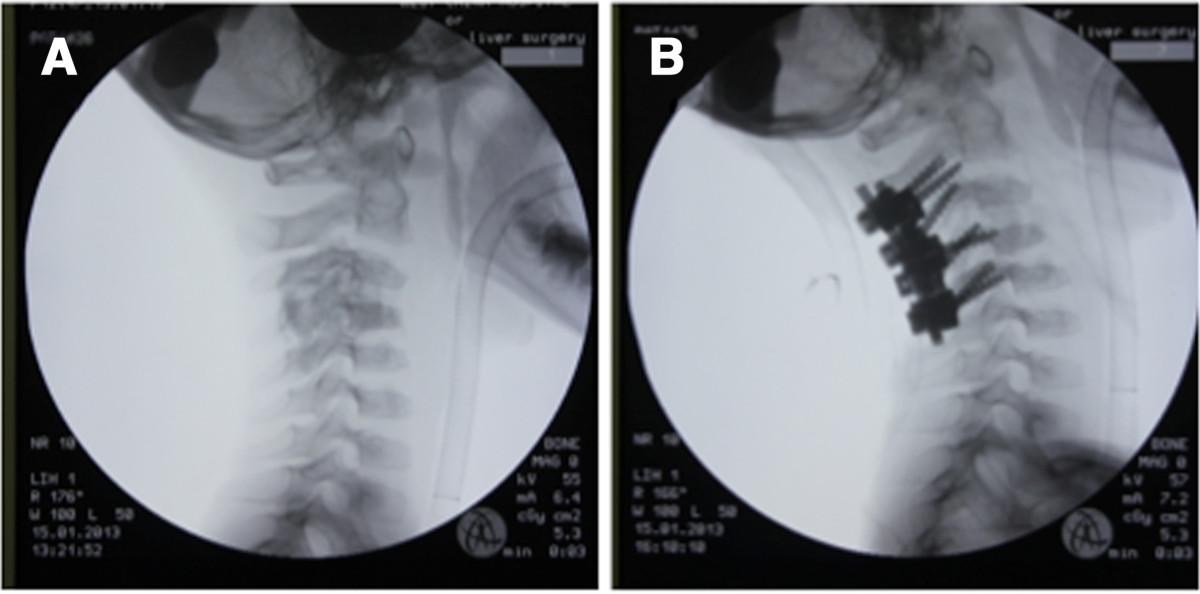
**Intraoperative radiograph shows no spinal instability after anesthesia was induced and the Mayfield clamp was positioned (A), and the result of the reconstructive technique (B).**

**Figure 5:**
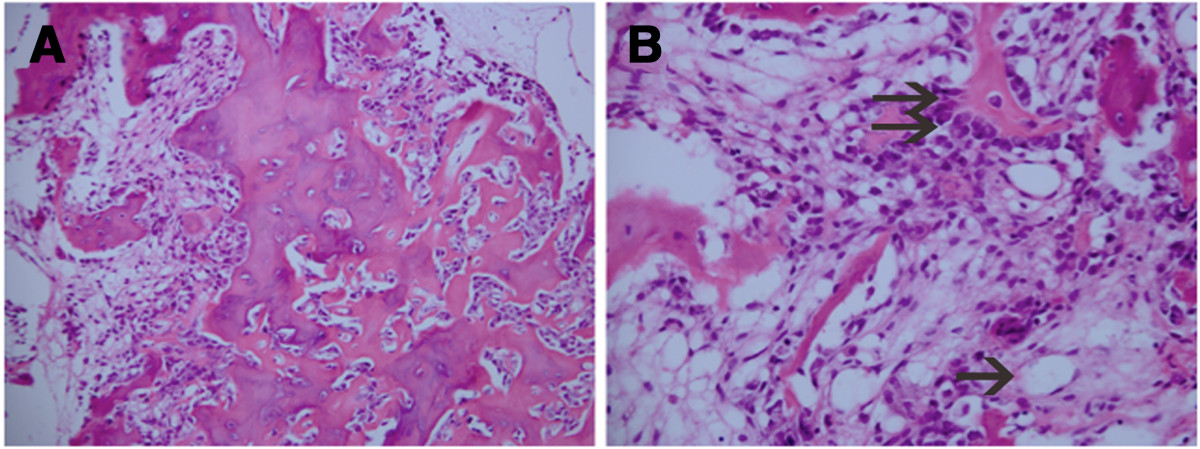
**Histology results. (A)**: Neoplastic, spindle-shaped cells within an interspersed osteoid network with bone formation and fibroconnective tissue (hematoxylin-eosin, original magnification × 200); **(B)** There are abundant capillaries in the tissue (arrow), and osteoblasts can also be observed (hematoxylin-eosin, original magnification × 400).

**Figure 6:**
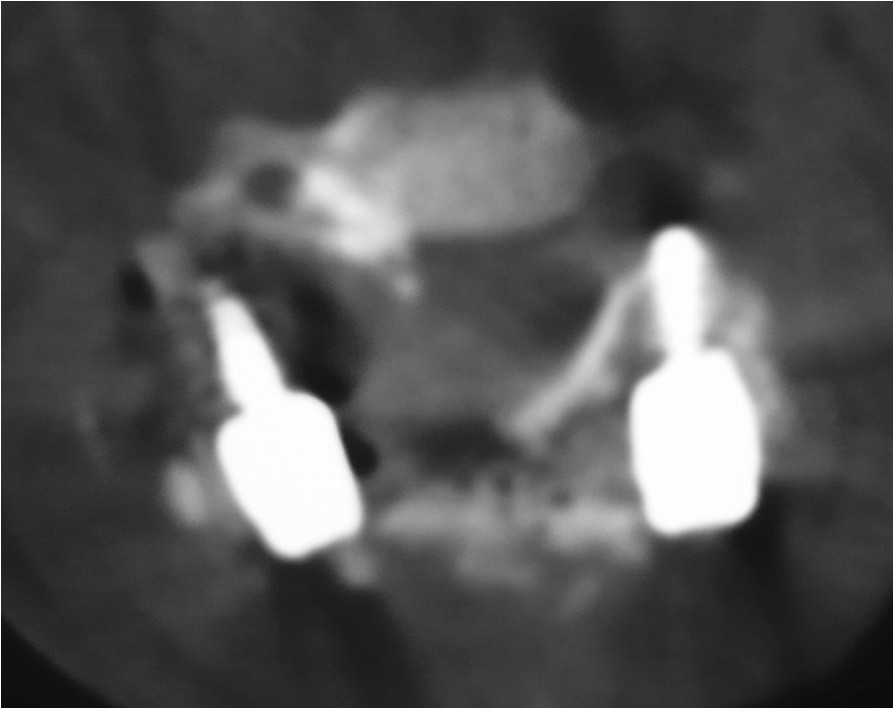
**At 1-year follow-up, the CT scan shows no evidence of tumor recurrence.**

After anesthesia, the patient was placed carefully in the prone position with a Mayfield 3-point head holder. Fluoroscopy confirmed that no iatrogenic subluxation or dislocation was caused by this position (Figure 4A). During the operation, a posterior approach was chosen and the lamina and articular complex of C2-C5 was exposed. A hemilaminectomy from C3 to C4 and facetectomy of the right side were performed. After laminectomy and facetectomy, the tumor was isolated. With a curette, the tumor was removed grossly and the spinal cord was decompressed. The circumferential margin of normal bony tissue was also removed with a high-speed burr. However, the right vertebral artery was injured during the extensive intralesional resection because of the close proximity of the tumor to the vertebral artery canal. The minor laceration of the vertebral artery was caused by the high-speed drill. During the drilling process, we observed a surge of bright red blood with rapid pulsatile flow. The area was quickly packed with the hemostatic, thrombin-soaked gelatin sponges and cottonoids. When complete hemostasis was obtained, a tricortical iliac crest was used to fill the bony gap left after the tumor mass excision and was fixed with a screw. The crest also added pressure to the gelatin sponges to prevent postoperative bleeding. Subsequently, the screw was connected with the upper and lower lateral mass screw-rod system (Figure 4B). The right-side facets were decorticated and fused to stabilize the spine.The pathologic examination of the surgical specimen indicated osteoblastoma (Figure 5A, B). The pain of the neck was relieved significantly postoperatively. The patient gradually recovered neurologic function. At the 12-month follow up, the patient presented full neurologic recovery and no evidence of recurrence by CT scan (Figure 6).

## Conclusion

A diagnostic delay is common in cases of cervical osteoblastoma. Symptoms of patients afflicted with osteoblastoma of the spine usually include dull, localized, and gradually increasing pain. However, these symptoms were non-specific and usually neglected by physicians, which may have been the cause of the 6-month diagnosis delay in the present case. The patient was eventually diagnosed correctly because of his severe neurological deficit and was managed surgically. Neurologic deficits are occasionally reported in the literature. In our case, the patient presented with muscle weakness, sensory disturbance, and hyperreflexia.

In some cases, the vertebral artery canal was eroded by the osteoblastoma, and the use of preoperative angiography and embolization was useful to reduce intraoperative blood loss [8]. However, preoperative angiography and embolization were not performed in our case because of economic limitations of the patient’s family. However, vascular complications during the operation in our case indicate the necessity of preoperative embolization.

There should be always an attempt to achieve diagnostics by biopsy before surgery, which has become a necessary prelude to successful management of suspected spinal tumors. In this case, the differential diagnosis with osteosarcoma and osteoma osteoid is quite unlikely before operation. In addition, biopsy is generally preferred for a variety of reasons, including lower morbidity, cost effectiveness and shorter procedure time and patient length of stay. However, we didn’t perform pre-operative biopsy in this case for the following reasons: (1) According to the literature, sclerotic lesions showed a low diagnostic accuracy rate by biopsy [9]. Specimens from sclerotic lesions are composed chiefly of eburnated bone containing only tiny areas of bone marrow with a few cell clusters; (2) Hemorrhage may occur during biopsy because of rich tumor vascularization; (3) The use of good quality radiological exams can fulfill a criterion for that particular histopathology. As the CT and MRI image was so suggestive of osteoblatoma, the biopsy was avoided.

This tumor was classified as benign lesion, locally aggressive, the treatment of which is ideally performed by a marginal resection. However, the surgical resection was planned to be intra-lesional resection because of the proximity of the noble anatomic structures.

On the 12-month follow-up CT, a considerable narrowing of the vertebral canal was observed, which was probably caused by insufficient tumor removal. Although a complete excision of the lesion was not achieved because of vertebral artery injury, the gross and microscopic tumor, as well as a circumferential margin of normal bony tissue was removed. At the 12-month follow-up, the patient presented without neurologic deficit, and disease progression or recurrence was not observed because of the insufficient removal.

Blindness after spinal surgery has been described as a rare but serious, irreversible, and incurable complication. Previously reports have described the delayed onset of cortical blindness secondary to vertebral artery injury following cervical spine trauma [10]. The visual dysfunction was probably caused by embolic cortical infarction superimposed on the vertebral artery dissection. However, the patient in our case is asymptomatic, and the eye examination was documented as normal. The possible explanation for the different outcome in our case may be the presence of collateral blood supply from the contralateral vertebral artery and circle of Willis.

Whether spinal fusion is necessary after tumor resection remains controversial. Fusion for cervical stabilization substantially decreases the mobility of the cervical spine and predisposes the discs above and below the fusion to degeneration. However, stabilization of the cervical spine is critical to prevent progressive kyphotic deformity. Several previous reports have described kyphotic deformity following intralesional excision of osteoblastoma [11–13]. Children and young adults (25 years or younger) are considered at higher risk of postlaminectomy kyphosis than older adults because of the skeletal immaturity and continued growth characteristic of those stages. A long-term follow-up is needed for our case to determine whether tumor recurrence or spinal deformity will occur after the surgical reconstruction.

In conclusion, this case delineates the difficulties in diagnosing this tumor, the challenges and issues encountered during the surgical management, and also the favorable prognosis after adequate treatment.

### Consent

Written informed consent was obtained from the parents of the young patient for the publication of this case report and any accompanying images. A copy of the written consent is available for review by the Editor of this journal. The human subjects Institutional Review Board of Sichuan University approved this study prospectively.

## Authors’ original submitted files for images

Below are the links to the authors’ original submitted files for images. Authors’ original file for figure 1 Authors’ original file for figure 2 Authors’ original file for figure 3 Authors’ original file for figure 4 Authors’ original file for figure 5 Authors’ original file for figure 6

## References

[1] Boriani S, Amendola L, Bandiera S, Simoes CE, Alberghini M, Di Fiore M, Gasbarrini A (2012). Staging and treatment of osteoblastoma in the mobile spine: a review of 51 cases. Eur Spine J.

[2] Harrop JS, Schmidt MH, Boriani S, Shaffrey CI (2009). Aggressive “benign” primary spine neoplasms: osteoblastoma, aneurysmal bone cyst, and giant cell tumor. Spine (Phila Pa 1976).

[3] Amirjamshidi A, Abbassioun K (2010). Osteoblastoma of the third cervical vertebra in a 16-year-old boy: case report and review of the literature. Pediatr Neurosurg.

[4] Burn SC, Ansorge O, Zeller R, Drake JM (2009). Management of osteoblastoma and osteoid osteoma of the spine in childhood. J Neurosurg Pediatr.

[5] Lian X, Zhao J, Hou T, Ma H, Chen Z (2006). Benign intraspinal osteoblastoma stemming from C7 lamina in cervicothoracic junction: a case report. Spine (Phila Pa 1976).

[6] Samdani A, Torre-Healy A, Chou D, Cahill AM, Storm PB (2009). Treatment of osteoblastoma at C7: a multidisciplinary approach. A case report and review of the literature. Eur Spine J.

[7] Chakrapani SD, Grim K, Kaimaktchiev V, Anderson JC (2008). Osteoblastoma of the spine with discordant magnetic resonance imaging and computed tomography imaging features in a child. Spine (Phila Pa 1976).

[8] Trubenbach J, Nagele T, Bauer T, Ernemann U (2006). Preoperative embolization of cervical spine osteoblastomas: report of three cases. AJNR Am J Neuroradiol.

[9] Vieillard MH, Boutry N, Chastanet P, Duquesnoy B, Cotten A, Cortet B (2005). Contribution of percutaneous biopsy to the definite diagnosis in patients with suspected bone tumor. Joint Bone Spine.

[10] Fassett DR, Dailey AT, Vaccaro AR (2008). Vertebral artery injuries associated with cervical spine injuries: a review of the literature. J Spinal Disord Tech.

[11] Deutsch H, Haid RW, Rodts GE, Mummaneni PV (2003). Postlaminectomy cervical deformity. Neurosurg Focus.

[12] Fassett DR, Clark R, Brockmeyer DL, Schmidt MH (2006). Cervical spine deformity associated with resection of spinal cord tumors. Neurosurg Focus.

[13] Albert TJ, Vacarro A (1998). Postlaminectomy kyphosis. Spine (Phila Pa 1976).

[CR14] The pre-publication history for this paper can be accessed here:http://www.biomedcentral.com/1471-2474/15/313/prepub

