# Supplementary figures and images for: Treatment of osteoblastoma at C3-4 in a child: a case report
Source: BMC Musculoskelet Disord. 2014 Sep 26;15:313. doi: 10.1186/1471-2474-15-313 (PMC4195949; doi:10.1186/1471-2474-15-313)

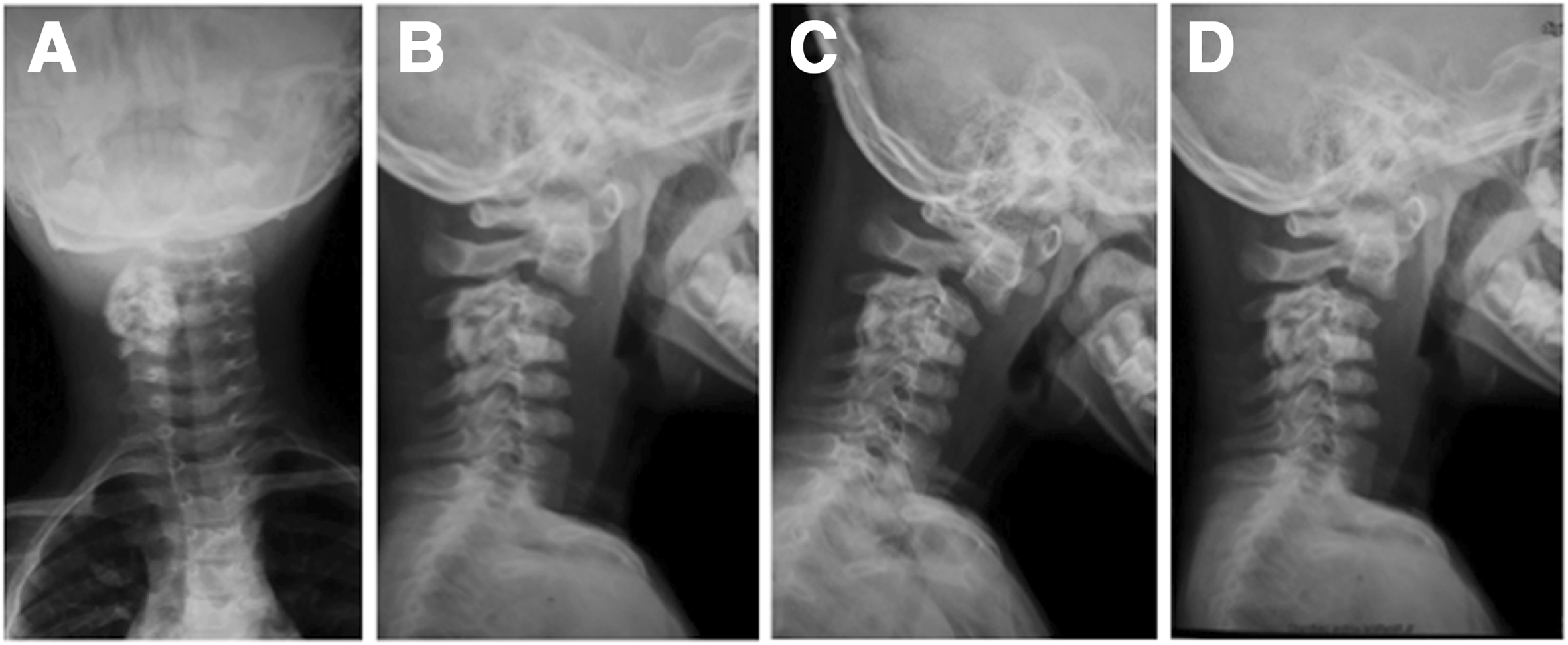

Supplement: Supplementary file 1 — Authors’ original file for figure 1 [file 12891_2014_2267_MOESM1_ESM.tif]

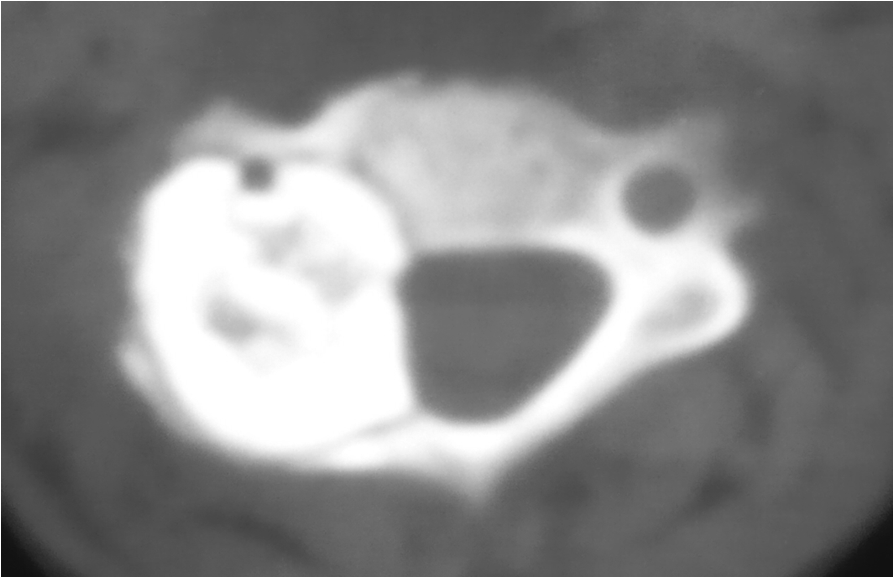

Supplement: Supplementary file 2 — Authors’ original file for figure 2 [file 12891_2014_2267_MOESM2_ESM.tif]

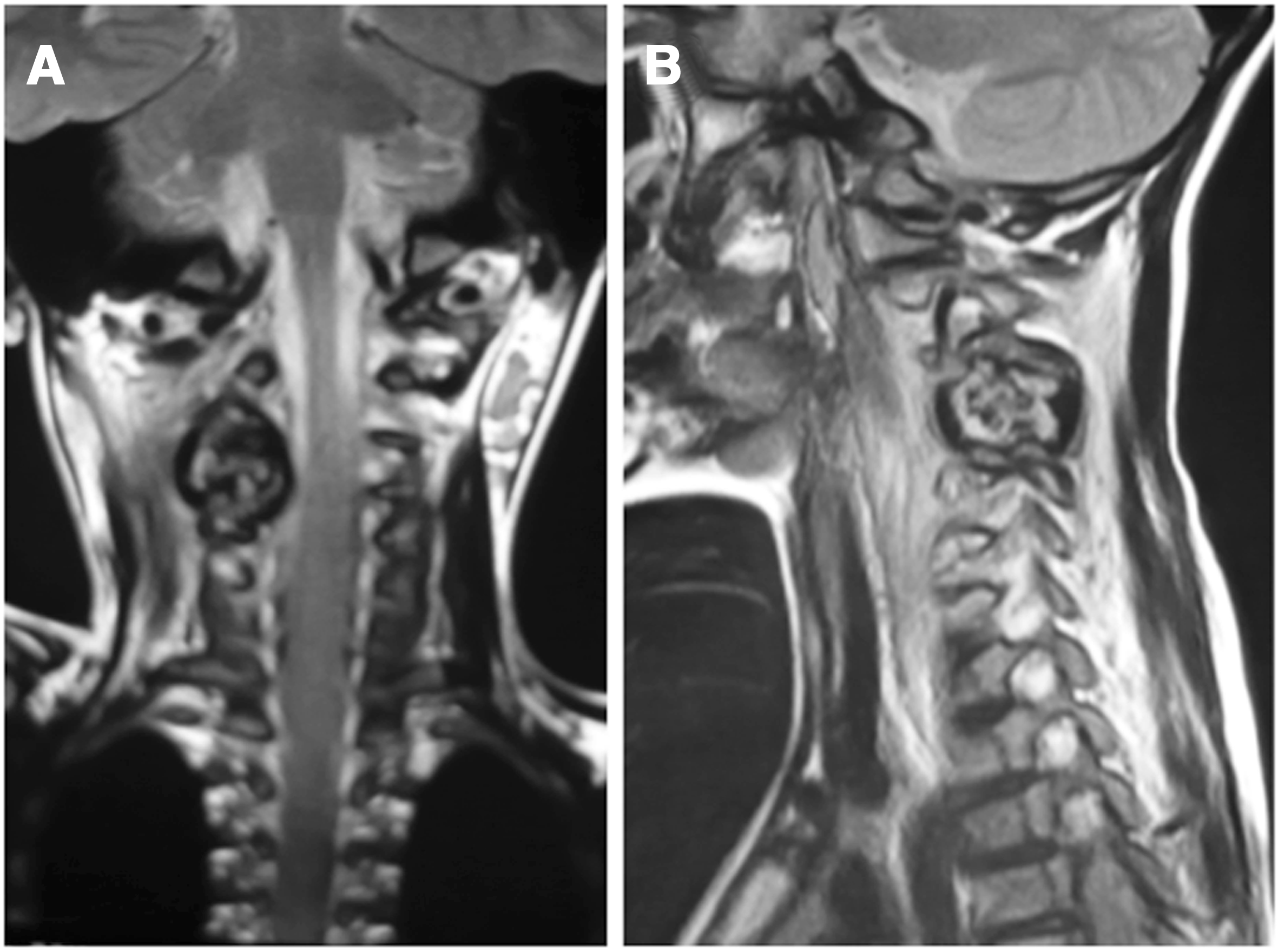

Supplement: Supplementary file 3 — Authors’ original file for figure 3 [file 12891_2014_2267_MOESM3_ESM.tif]

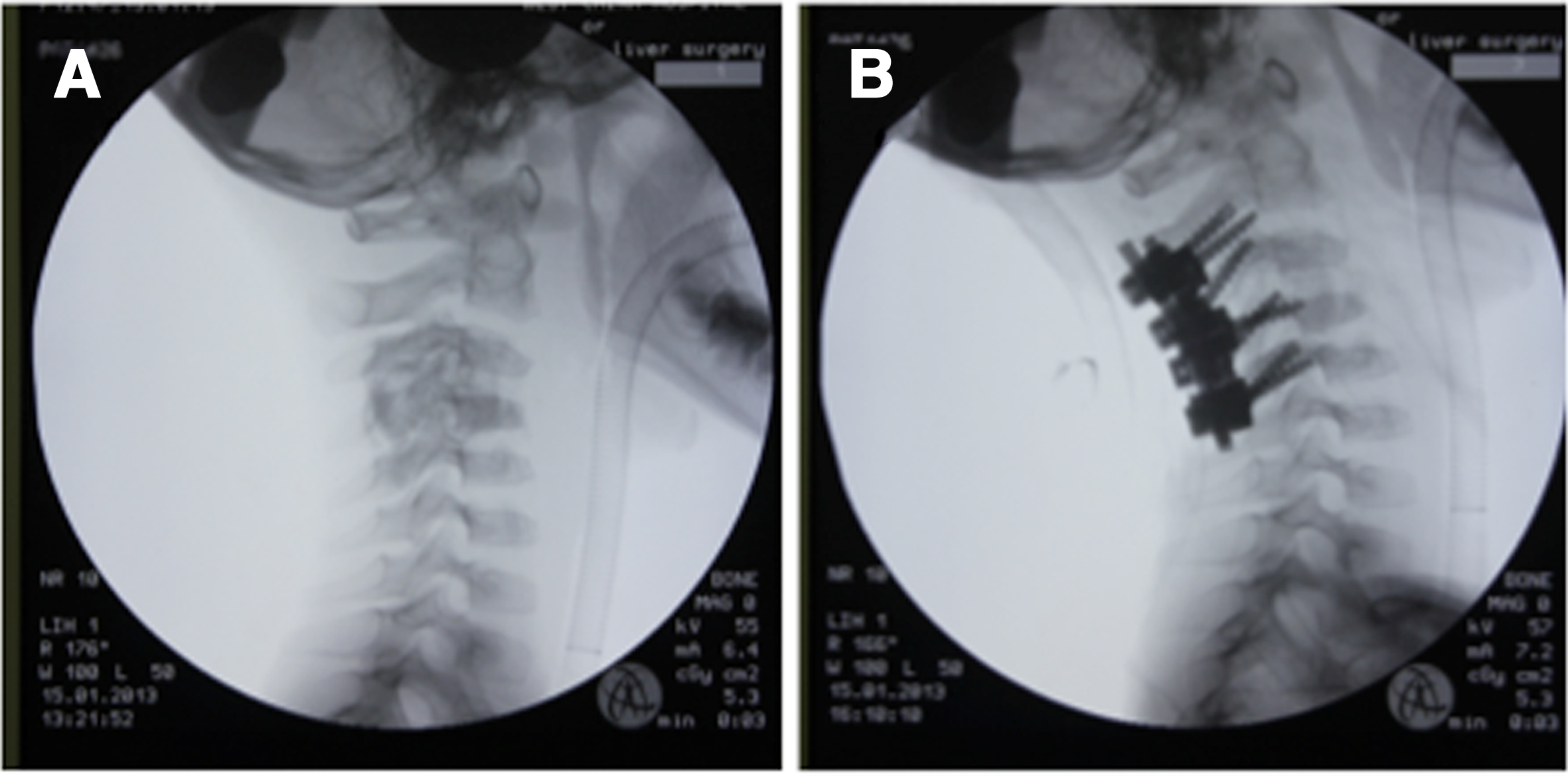

Supplement: Supplementary file 4 — Authors’ original file for figure 4 [file 12891_2014_2267_MOESM4_ESM.tif]

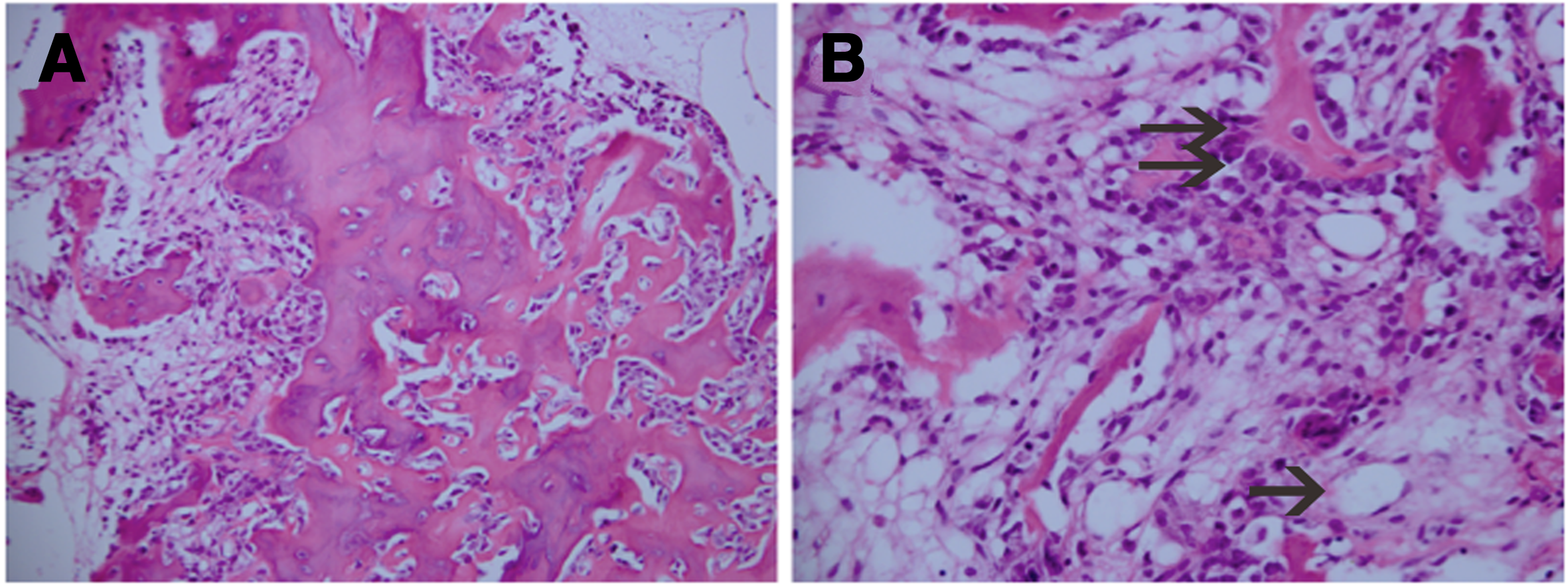

Supplement: Supplementary file 5 — Authors’ original file for figure 5 [file 12891_2014_2267_MOESM5_ESM.tif]

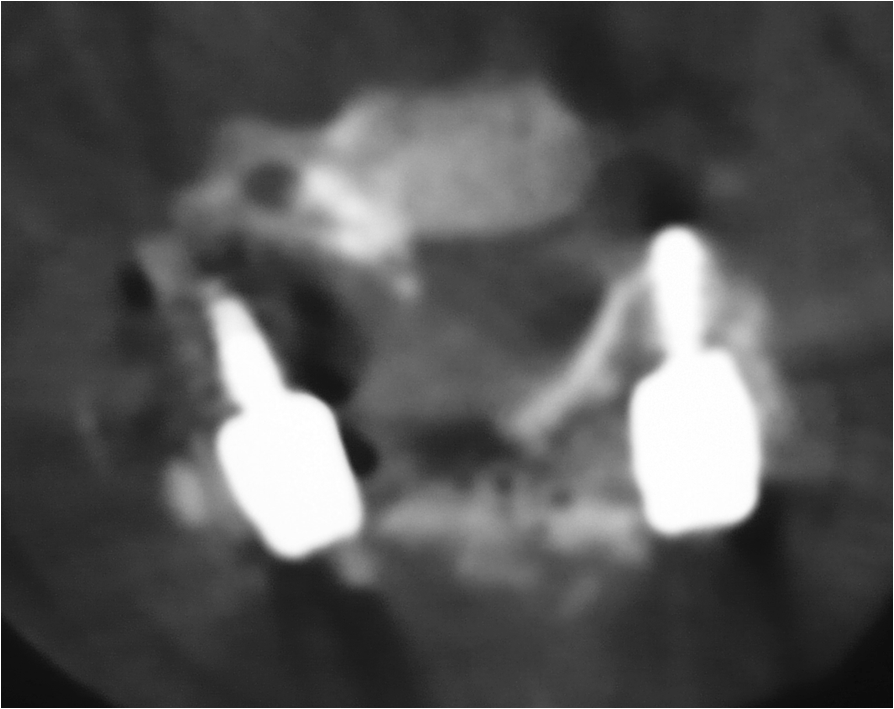

Supplement: Supplementary file 6 — Authors’ original file for figure 6 [file 12891_2014_2267_MOESM6_ESM.tif]
